# ETV5-mediated upregulation of lncRNA CTBP1-DT as a ceRNA facilitates HGSOC progression by regulating miR-188-5p/MAP3K3 axis

**DOI:** 10.1038/s41419-021-04256-9

**Published:** 2021-12-09

**Authors:** Ping Liu, Ruiting Fu, Kai Chen, Lu Zhang, Shasha Wang, Weihua Liang, Hong Zou, Lin Tao, Wei Jia

**Affiliations:** 1 NHC Key Laboratory of Prevention and Treatment of Central Asia High Incidence Diseases, First Affiliated Hospital, School of Medicine, Shihezi University/Department of Pathology and Key Laboratory for Xinjiang Endemic and Ethnic Diseases, Shihezi University School of Medicine, Shihezi, China; 2 Department of Pathology and Key Laboratory for Xinjiang Endemic and Ethnic Diseases, Shihezi University School of Medicine, Shihezi, China; 3 Department of Obstetrics and Gynecology, First Affiliated Hospital, School of Medicine, Shihezi University, Shihezi, China; 4 Department of Pathology, Binzhou Medical University Hospital, Binzhou, China

**Keywords:** Ovarian cancer, Oncogenes

## Abstract

High-grade serous ovarian cancer (HGSOC) is a common and lethal cancer of the female reproductive system. Long non-coding RNAs (lncRNAs) are aberrantly expressed in various cancers and play crucial roles in tumour progression. However, their function and molecular mechanism in HGSOC remain largely unknown. Based on public databases and bioinformatics analyses, the overexpression of lncRNA CTBP1-DT in HGSOC tissues was detected and validated in a cohort of HGSOC tissues. High expression of lncRNA CTBP1-DT was associated with poor prognosis and was an independent risk factor for survival. Overexpression of lncRNA CTBP1-DT promoted malignant biological behaviour of HGSOC cells, whereas its depletion induced growth arrest of HGSOC cells by vitro and in vivo assays. Mechanistically, lncRNA CTBP1-DT could competitively bind to miR-188-5p to protect MAP3K3 from degradation. Moreover, our results revealed that ETV5 could specifically interact with the promoter of lncRNA CTBP1-DT and activate its transcription. Collectively, these results reveal a novel ETV5/lncRNA CTBP1-DT/miR-188-5p/MAP3K3 pathway for HGSOC progression and suggest that lncRNA CTBP1-DT might be a potential biomarker and therapeutic target for HGSOC.

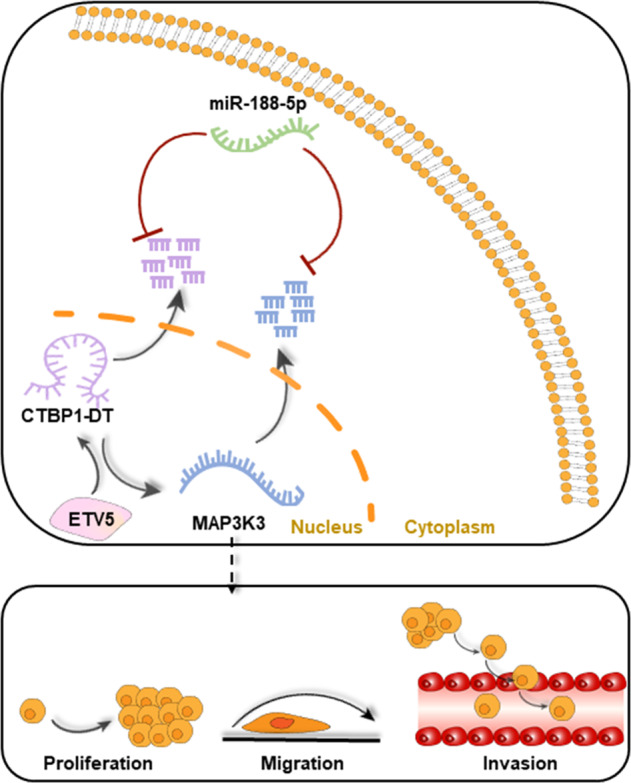

## Introduction

The global burden of ovarian cancer (OC) accounts for 2.5% of all malignancies among women [1]. It is the seventh most diagnosed cancer among women worldwide. In 2021, approximately 21,410 new diagnoses of OC and 13,770 OC deaths were expected to occur in the United States [2]. High-grade serous ovarian cancer (HGSOC) is the most common histological subtype of OC, accounting for 67.5% of OC subtypes [1, 3]. HGSOC is usually diagnosed at advanced stages due to the absence of characteristic symptoms at early stages [4]. It involves both ovaries and is characterised by aggressive behaviour and low survival. Despite the continuous improvement and development of surgical and systematic treatment, the mortality of OC patients has only decreased slightly. To date, the treatment of OC is still based on traditional surgery combined with chemotherapy. Under this treatment scheme, nearly 70% of patients experience relapse and chemotherapy drug resistance after treatment, and compared with other tumours, OC lacks effective molecular targeted therapy [3, 5]. Therefore, there is a pressing need to identify key molecular events underlying the progression and metastasis of HGSOC to improve diagnosis and prognosis.

Long non-coding RNAs (lncRNAs) are a set of non-protein-coding transcripts longer than 200 nucleotides maintained at relatively low expression levels. They are involved in multiple biological processes, including post-transcriptional regulation, organisation of protein complexes, cell-cell signalling and allosteric regulation of proteins [6, 7]. LncRNAs play crucial roles in different carcinomas, including breast [8], colorectal [9], gastric [10] and renal [11] carcinoma. Furthermore, lncRNAs have been identified as novel biomarkers of many carcinomas [12, 13]. LncRNA CTBP1-DT, a newly identified lncRNA, regulates cervical cancer progression by sponging miR-3163 to upregulate ZNF217 [14], which could promote cell proliferation in hepatocellular carcinoma by regulating the miR-623/cyclin D1 axis [15]. However, the clinical significance and underlying biological mechanisms of lncRNA CTBP1-DT in the regulation of HGSOC have not been elucidated.

LncRNAs and mRNAs could interact by competing with microRNAs (miRNAs) [16, 17]. LncRNAs can also function as competing endogenous RNAs (ceRNAs) by competitively binding miRNAs, thereby modulating the de-repression of miRNA targets in the cytoplasm [18, 19]. For example, lncRNA PTAR, which may serve as a competing endogenous lncRNA by sponging miR-101, mediates the role of transforming growth factor β-induced epithelial-mesenchymal transition and the invasion-metastasis cascade of OC [20]. Thus, this provides new insight into the role of ncRNAs in tumours. Our previous study indicated that MAP3K3 was overexpressed and continuously activated nuclear factor kappa B signalling in HGSOC; it is also an independent prognostic factor for patients with HGSOC [21, 22]. LncRNA CTBP1-DT, screened from The Cancer Genome Atlas (TCGA) database, was positively correlated with MAP3K3 expression. Whether lncRNA CTBP1-DT could regulate MAP3K3 to influence the biological behaviours of HGSOC cells is not clear.

In the present study, we discovered that lncRNA CTBP1-DT is significantly overexpressed in HGSOC tissues and is associated with poor prognosis of HGSOC patients. It functions as a key regulator by competitively binding to miR-188-5p to protect MAP3K3 from degradation and thus promotes the growth and progression of HGSOC both in vitro and in vivo. In addition, we observed that ETS variant transcription factor 5 (ETV5)-mediated transcription of lncRNA CTBP1-DT consequently facilitates the malignant biological behaviour of HGSOC cells. Our results provide novel insights into the progression and metastasis of HGSOC and a promising therapeutic target for HGSOC treatment.

## Results

### LncRNA CTBP1-DT is highly expressed in HGSOC and correlates with poor prognosis

**Fig. 1:**
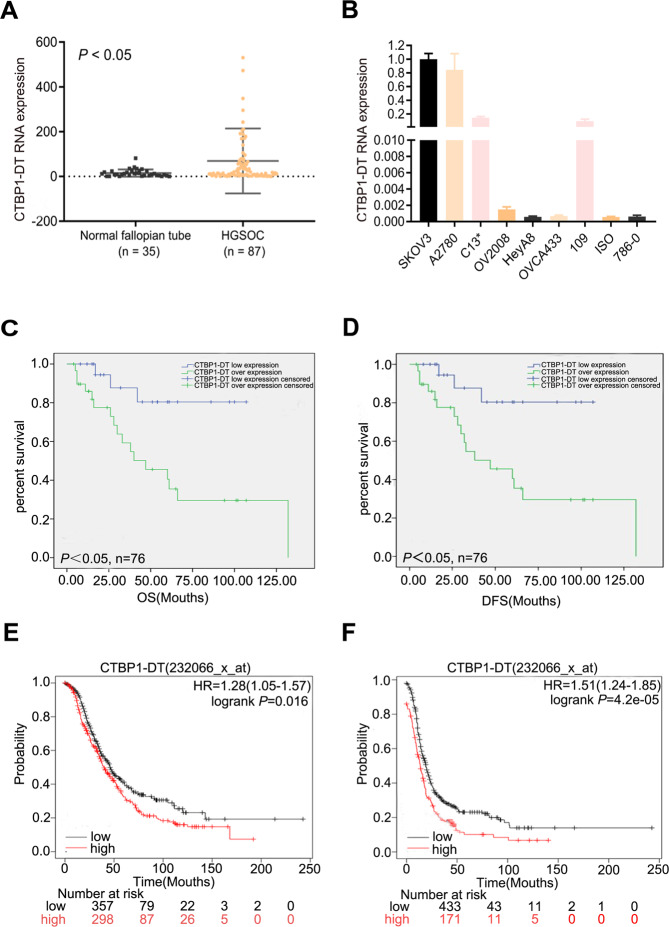
LncRNA CTBP1-DT is upregulated in HGSOC cells and positively correlates with poor prognosis in HGSOC patients. **A** The expression of lncRNA CTBP1-DT was detected in 87 HGSOC tissues and 35 normal fallopian tube by qRT-PCR. **B** The relative expression of lncRNA CTBP1-DT in ovarian carcinoma cell lines (SKOV3, A2780, C13*, OV2008, HeyA8 and OVCA433), oesophageal carcinoma cells (109), angiosarcoma cells (ISO) and renal clear cell carcinoma cells (786–0) was detected by qRT-PCR. **C**, **D** Kaplan–Meier plots were used to determine the association between the lncRNA CTBP1-DT expression level and the OS/DFS of patients with HGSOC. **E**, **F** Log-rank test was used to determine the association between lncRNA CTBP1-DT expression level and the survival times in patients with HGSOC in the TCGA HGSOC datasets. Data are presented as the mean ± SEM. *P < 0.05, **P < 0.01, ***P < 0.001.

To investigate the role of lncRNA CTBP1-DT in human HGSOC, lncRNA CTBP1-DT expression was determined in 87 HGSOC tissues and 35 normal fallopian tube tissues; it was significantly overexpressed in HGSOC tissues than in normal fallopian tube tissues (Fig. 1A). LncRNA CTBP1-DT expression was relatively higher in ovarian carcinoma cell lines (SKOV3, A2780, C13*, OV2008, HeyA8 and OVCA433) than in oesophageal carcinoma cells (109), angiosarcoma cells (ISO) and clear cell renal cell carcinoma cells (786–0) (Fig. 1B).

The correlation of lncRNA CTBP1-DT expression with clinicopathological factors in HGSOC patients is summarised in Table 1. LncRNA CTBP1-DT overexpression was significantly associated with FIGO stage (*P* = 0.044), age (*P* = 0.006) and chemotherapeutic response (*P* = 0.027). Survival information of the registered 76 patients was gathered, and overall survival (OS) and disease-free survival (DFS) curves were obtained using the Kaplan–Meier assay. Patients with lncRNA CTBP1-DT overexpression had significantly shorter OS and DFS rates (*P* < 0.05, Fig. 1C, D). Similarly, higher lncRNA CTBP1-DT expression correlated with significantly shorter OS and DFS in the TCGA HGSOC dataset (*P* < 0.05, Fig. 1E, F).

**Table 1:** The correlation of lncRNA CTBP1-DT expression with clinicopathological factors of HGSOC patients.

Characteristics	*n*	CTBP1-DT expression		*X*^2^	*P* value
	87	Overexpression (%)	Low expression (%)		
*Age*					
<55	44	20 (45.5)	24 (54.5)	7.587	0.006**
≥55	43	32 (74.4)	11 (25.6)		
*FIGO stage*					
I–II	21	12 (57.1)	9 (42.9)	4.066	0.044*
III–IV	66	40 (60.6)	26 (39.4)		
*Chemotherapy response*					
Sensitive	34	15 (44.1)	19 (55.9)	4.870	0.027*
Partial	34	24 (70.6)	10 (29.4)		
Unknown	19				
*Ascites*					
No	26	16 (61.5)	10 (38.5)	0.048	0.826
Yes	61	36 (59.0)	25 (41.0)		

*FIGO* Federation International of Gynecology and Obstetrics, *HGSOC* high-grade serous ovarian cancer.

LncRNA CTBP1-DT overexpression was significantly associated with age (*P* = 0.006), FIGO stage (*P* = 0.044) and chemotherapy response (*P* = 0.027) by *χ*^2^ test. *P < 0.05, **P < 0.01.

Moreover, univariate analysis of the Cox regression model indicated that expression of lncRNA CTBP1-DT (*P* = 0.048), age (*P* = 0.048) and FIGO stage (*P* = 0.006) might be the prognostic factors for patients with HGSOC. Multivariate analysis showed that FIGO stage was an independent risk factor for HGSOC (Table 2). These results indicated that lncRNA CTBP1-DT expression was positively associated with poor prognosis of HGSOC patients.

**Table 2:** Univariate and multivariate analyses of the associations between HGSOC patient risk factors.

	Univariate		Multivariate	
	HR (95% CI)	*P* value	HR (95% CI)	*P* value
CTBP1-DT (low vs overexpression)	2.155 (1.008–4.607)	0.048*	1.718 (0.796–3.710)	0.168
Age (<55 vs ≥55)	2.005 (1.008–3.988)	0.048*	1.983 (0.972–4.043)	0.060
FIGO stage (I–II vs III–IV)	5.420 (1.615–18.186)	0.006**	5.780 (1.663–20.089)	0.006**
Chemotherapy response (sensitive vs partial)	0.887 (0.358–2.201)	0.976		
Ascites (no vs yes)	1.247 (0.610–2.552)	1.247		

*CI* confidence interval, *FIGO* Federation International of Gynecology and Obstetrics, *HR* hazard ratio.

Expression of lncRNA CTBP1-DT (*P* = 0.048), age (*P* = 0.048) and FIGO stage (*P* = 0.006) might be the prognostic factors for patients with HGSOC by univariate analysis of the Cox regression model. FIGO stage (*P* = 0.006) was an independent risk factor for patients with HGSOC by multivariate analysis. *P < 0.05, **P < 0.01.

### LncRNA CTBP1-DT promotes HGSOC cell proliferation, migration and invasion in vivo and in vitro

**Fig. 2:**
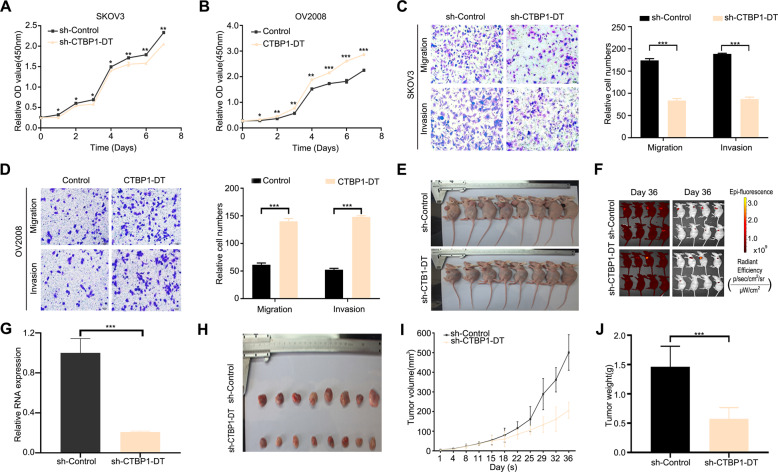
LncRNA CTBP1-DT promotes HGSOC cell proliferation, migration and invasion in vivo and in vitro. **A**, **B** CCK-8 assays were performed to determine the proliferation ability of SKOV3 cells transfected with sh-lncRNA CTBP1-DT or NC and OV2008 cells transfected with lncRNA CTBP1-DT or vector. **C**, **D** Transwell assays were performed in SKOV3 cells transfected with sh-lncRNA CTBP1-DT or control and OV2008 cells transfected with lncRNA CTBP1-DT or vector to explore cell migratory and invasive capabilities. **E** Image of mice in the lncRNA CTBP1-DT-knockdown and control groups. **F** Image of mice in the lncRNA CTBP1-DT-knockdown and control groups by in vivo optical imaging with bioluminescence and fluorescence at day 36. **G** qRT-PCR was applied to determine lncRNA CTBP1-DT expression in tissues derived from the xenograft tumour model. **H** Image of subcutaneous tumour tissues in the lncRNA CTBP1-DT-overexpression group and control group. **I** Analysis of tumour volume of mice. **J** The relative weights of mice tumours were measured. Data are reported as mean ± SEM. *P < 0.05, **P < 0.01, ***P < 0.001.

The functional effects of lncRNA CTBP1-DT in HGSOC were explored. Functionally, the cell proliferation assay demonstrated that knockdown of lncRNA CTBP1-DT significantly decreased cell proliferation in SKOV3 cells, whereas overexpression of lncRNA CTBP1-DT significantly increased cell proliferation in OV2008 cells (Fig. 2A, B). We further investigated the role of lncRNA CTBP1-DT in the motility of HGSOC cells. As shown in Fig. 2C, the transfection of lncRNA CTBP1-DT with short hairpin RNA (shRNA) impeded the migratory ability of SKOV3 cells. A corresponding effect on invasiveness was also observed in a parallel invasion assay. Conversely, transfection of OV2008 cells with the lncRNA CTBP1-DT vector promoted cell migration and invasiveness (Fig. 2D). These results indicate that lncRNA CTBP1-DT has oncogenic properties that can promote the migratory and invasive phenotype in HGSOC cells.

To clarify the effects of lncRNA CTBP1-DT on tumour growth in vivo, SKOV3 cells with stable lncRNA CTBP1-DT shRNA were subcutaneously injected into nude mice (Fig. 2E). In vivo optical imaging with bioluminescence and fluorescence was performed to detect the metastatic effect of sh-lncRNA CTBP1-DT in vivo. Unfortunately, we did not find any metastatic nodules of the transplanted tumours (Fig. 2F). Even after autopsy, non-metastatic nodules were found in the abdominal cavity, greater omentum, liver, lung and other organs. Quantitative reverse transcription polymerase chain reaction (qRT-PCR) assays showed that the expression of lncRNA CTBP1-DT was markedly decreased in xenograft tumours from the sh-lncRNA CTBP1-DT mice (Fig. 2G). Consistent with the in vitro results, low expression of lncRNA CTBP1-DT significantly reduced the tumour volume and weight compared with those in the control group (Fig. 2H–J).

### LncRNA CTBP1-DT promotes the malignant biological behaviour of HGSOC cells by upregulating MAP3K3

**Fig. 3:**
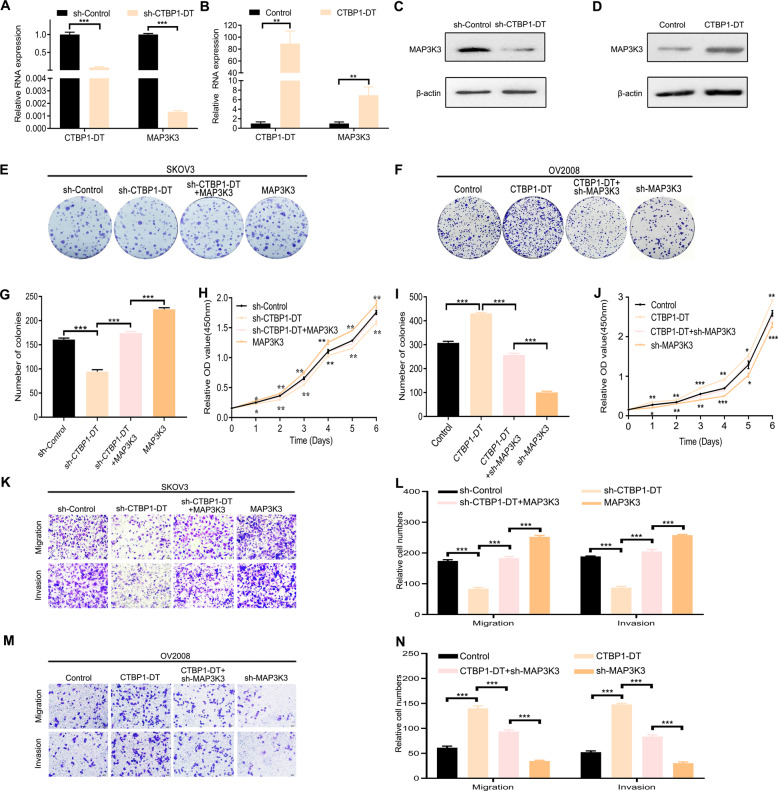
LncRNA CTBP1-DT promotes the malignant biological behaviour of HGSOC cells by upregulating MAP3K3. **A**, **B** LncRNA CTBP1-DT knockdown resulted in decreased expression of MAP3K3 in SKOV3 cells, lncRNA CTBP1-DT overexpression resulted in increased expression of MAP3K3 in OV2008 cells. **C**, **D** The efficiency of lncRNA CTBP1-DT knockdown or overexpression was detected by western blot assay in SKOV3 and OV2008. **E**, **G**, **H** CCK-8 assays and colony formation assays were performed to determine the proliferation ability of SKOV3 cells transfected with NC, sh-lncRNA CTBP1-DT, MAP3K3 and sh-lncRNA CTBP1-DT with MAP3K3, respectively. **F**, **I**, **J** The proliferation ability was detected by CCK-8 assays and colony formation assays of OV2008 cells transfected with NC, lncRNA CTBP1-DT, sh-MAP3K3 and lncRNA CTBP1-DT with sh-MAP3K3. **K**, **L** Migration and invasion assays of SKOV3 cells with lncRNA CTBP1-DT knockdown and MAP3K3 overexpression. **M**, **N** Migration and invasion assays of OV2008 cells with lncRNA CTBP1-DT overexpression and MAP3K3 knockdown. Data are presented as the mean ± SEM. *P < 0.05, **P < 0.01, ***P < 0.001.

To clarify the mechanism and biological function of lncRNA CTBP1-DT in HGSOC cells, bioinformatics analysis revealed that overexpression of lncRNA CTBP1-DT corresponded with overexpression of MAP3K3 in HGSOC. Moreover, we verified the correlation between MAP3K3 and lncRNA CTBP1-DT. The abundance of MAP3K3 mRNA and protein in SKOV3 cells with endogenous overexpression of MAP3K3 decreased after knockdown of lncRNA CTBP1-DT (Fig. 3A, C). Conversely, the transfection of lncRNA CTBP1-DT enhanced the abundance of MAP3K3 in OV2008 cells (Fig. 3B, D).

Knockdown of lncRNA CTBP1-DT in SKOV3 cells inhibited cell proliferation, migration and invasion, which could be reversed by overexpression of MAP3K3 (Fig. 3E, G, H, K, L). In contrast, overexpression of lncRNA CTBP1-DT promoted proliferation, migration and invasion of OV2008 cells, which could be reversed by knockdown of MAP3K3 (Fig. 3F, I, J, M, N). Collectively, lncRNA CTBP1-DT could promote the malignant biological behaviour of HGSOC cells by upregulating the expression of MAP3K3.

### LncRNA CTBP1-DT serves as a miRNA sponge of miR-188-5p to regulate MAP3K3 expression in HGSOC cells

**Fig. 4:**
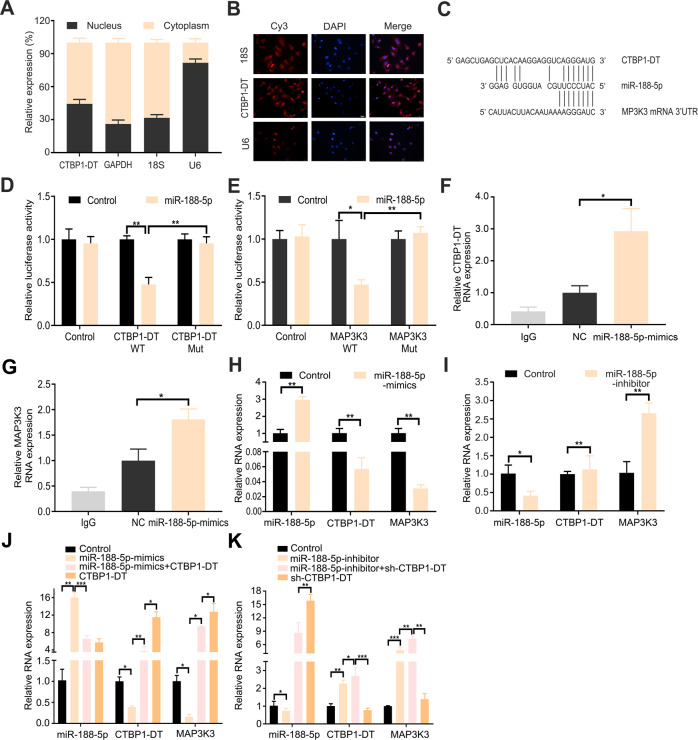
LncRNA CTBP1-DT acts as a miRNA sponge of miR-188-5p to regulate MAP3K3 expression in HGSOC cells. **A** qRT-PCR analysis for lncRNA CTBP1-DT expression and GAPDH, U6, 18S. The nuclear and cytoplasmic fractions of HEK 293T cells were located, followed by qRT-PCR assays. U6, 18S and GAPDH acted as negative controls. **B** The cellular location of lncRNA CTBP1-DT in cells by FISH assay (scale bar, 100 μm). **C** Schematic diagram representing the predicted binding sites for miR-188-5p, lncRNA CTBP1-DT and MAP3K3. **D**, **E** Firefly luciferase activity normalised to luciferase activity in HEK 293T cells co-transfected with luciferase reporters with wild-type or mutant transcripts of lncRNA CTBP1-DT or MAP3K3 along with miR-188-5p mimics or negative control. **F**, **G** RIP and RT-PCR were performed in HEK 293T cells to measure the lncRNA CTBP1-DT or MAP3K3 expression associated with miR-188-5p mimic. **H**, **I** miR-188-5p mimic overexpression resulted in decreased expression of lncRNA CTBP1-DT and MAP3K3, and miR-188-5p inhibitor overexpression upregulated lncRNA CTBP1-DT and MAP3K3 expression. **J** mRNA expression of lncRNA CTBP1-DT and MAP3K3 was determined by analysis after co-transfection with miR-188-5p mimic and lncRNA CTBP1-DT. **K** mRNA expression of lncRNA CTBP1-DT and MAP3K3 was determined by analysis after co-transfection with miR-188-5p inhibitor and sh-lncRNA CTBP1-DT. Data are presented as the mean ± SEM. *P < 0.05, **P < 0.01, ***P < 0.001.

LncRNA CTBP1-DT was distributed in both the cytoplasm and nucleus of SKOV3 cells (Fig. 4A, B). We hypothesised that lncRNA CTBP1-DT in the cell cytoplasm might act as a miRNA sponge to prevent miRNAs from binding MAP3K3. Through bioinformatics analysis, miR-188-5p could bind both the lncRNA CTBP1-DT and MAP3K3 in similar seed regions (Fig. 4C). Through luciferase assay, miR-188-5p inhibited the luciferase activities of the lncRNA CTBP1-DT wild-type binding motif, but there were no prominent differences in the mutant lncRNA CTBP1-DT (Fig. 4D). In addition, the luciferase activities of MAP3K3 wild-type binding sites were inhibited by miR-188-5p (Fig. 4E). These results indicated that miR-188-5p could interact with lncRNA CTBP1-DT and MAP3K3.

Subsequently, a binding RNA immunoprecipitation (RIP) assay was performed to validate their binding potential (Fig. 4F, G). The miR-188-5p inhibitor promoted lncRNA CTBP1-DT expression in OV2008 cells, whereas miR-188-5p mimics inhibited its expression in SKOV3. The expression of MAP3K3 corresponded with the expression of lncRNA CTBP1-DT (Fig. 4H, I). Consistent with these results, transfected miR-188-5p mimic reduced the expression of lncRNA CTBP1-DT and MAP3K3 in SKOV3. Moreover, MAP3K3 expression was reversed by overexpression of lncRNA CTBP1-DT (Fig. 4J). Transfection of the miR-188-5p inhibitor resulted in the upregulation of lncRNA CTBP1-DT and upregulation of MAP3K3, which were reversed after knockdown of lncRNA CTBP1-DT in OV2008 (Fig. 4K). Overall, in HGSOC cells, lncRNA CTBP1-DT serves as a miRNA sponge of miR-188-5p to regulate MAP3K3 expression.

### LncRNA CTBP1-DT is regulated by the transcription factor ETV5

**Fig. 5:**
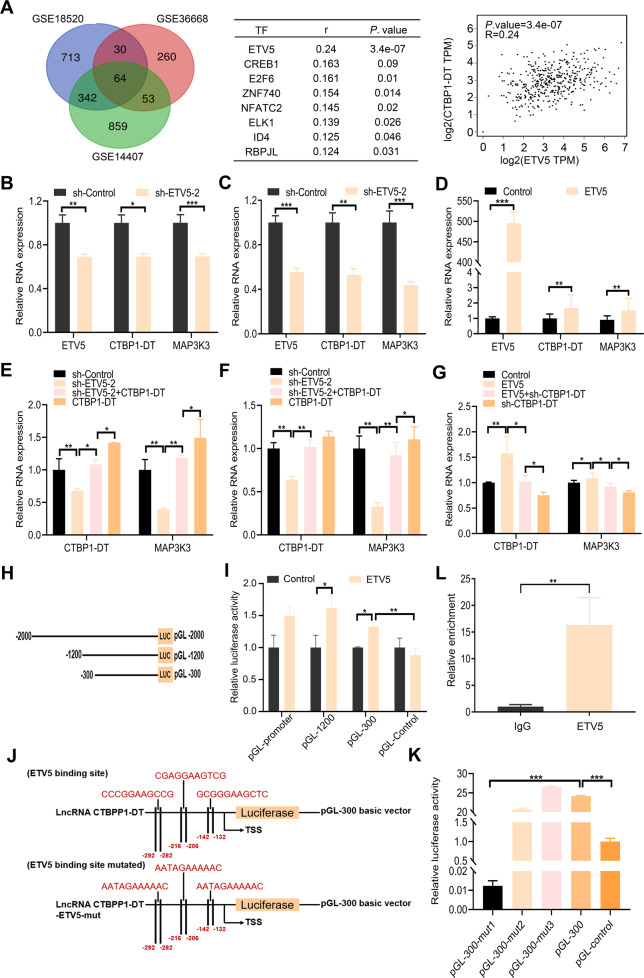
LncRNA CTBP1-DT is regulated by the transcription factor ETV5. **A** Using TCGA and JASPAR database, eight transcription factors were predicted to bind the promoter region of lncRNA CTBP1-DT. Among them, ETV5 had the highest correlation with lncRNA CTBP1-DT. **B**–**D** Expression of lncRNA CTBP1-DT in SKOV3 and A2780 cells after knockdown of ETV5 and in OV2008 cells after overexpression of ETV5. **E**–**G** The RNA expression of lncRNA CTBP1-DT and MAP3K3 in SKOV3, A2780 and OV2008 cells. **H** Schematic diagram of the lncRNA CTBP1-DT promoter fragments cloned into pGL0-basic vector upstream of the luciferase reporter gene, spanning from −2000/−1200/−300 to 0. **I** Luciferase assay of three truncated constructs was performed in HEK 293T cells. **J** Diagrammatic sketch of the luciferase reporter consisting of the lncRNA CTBP1-DT promoter. Schematic diagram of the luciferase reporter the mutant vector(mut-1/2/3). They included the basic promoter of hypothetical lncRNA CTBP1-DT binding site. **K** Luciferase activity of the lncRNA CTBPI-DT promoter was reduced when the three presumed ETVC lncRNA CTBP1-DT binding sites were imputed. **L** ChIP assay in HGSOC cells, followed by quantitative PCR amplification of the ETV5-binding site within the lncRNA CTBP1-DT promoter region. Quantitative PCR amplification explored bind site within lncRNA CTBP1-DT promoter region in HGSOC cells by ChIP assay. Data are presented as the mean ± SEM. *P < 0.05, **P < 0.01, ***P < 0.001.

To explore the mechanism of lncRNA CTBP1-DT upregulation in HGSOC, we applied the online bioinformatics software, JASPAR (http://jaspar.genereg.net/), to analyse transcription factors that could bind the promoter region of lncRNA CTBP1-DT. A total of eight potential transcription factors were found. Among them, ETV5 showed the highest correlation with lncRNA CTBP1-DT (Fig. 5A). We designed shRNAs targeting ETV5. Expression of lncRNA CTBP1-DT was measured following ETV5 knockdown or overexpression. The results revealed that ETV5 knockdown led to a significant decrease in the expression of lncRNA CTBP1-DT in SKOV3 and A2780 cells. Moreover, ETV5 overexpression enhanced the expression of lncRNA CTBP1-DT in OV2008 cells. The expression of MAP3K3 corresponded with that of lncRNA CTBP1-DT (Fig. 5B–D). Consistent with these results, we found that the knockdown of ETV5-2 decreased the expression of lncRNA CTBP1-DT and MAP3K3 in SKOV3 and A2780 cells. However, these effects were reversed by overexpression of lncRNA CTBP1-DT. Overexpression of ETV5 raised the expression of lncRNA CTBP1-DT and MAP3K3 in SKOV3 and A2780. However, these effects were reversed by knockdown of lncRNA CTBP1-DT (Fig. 5E–G).

To confirm that lncRNA CTBP1-DT was the transcriptional target of ETV5, we constructed three different deletion fragments of lncRNA CTBP1-DT, which were cloned into vectors (Fig. 5H). Luciferase activity was measured after transfection of these fragments of lncRNA CTBP1-DT into HEK 293T cells. Figure 5I shows that there was no statistically significant difference in luciferase activity between the full-length plasmid and the deletion constructs, indicating that 0–300-base pair (bp) fragments contained regulatory elements, which were critical for the transcription of lncRNA CTBP1-DT. We introduced three points mutations, designated as pGL-300-mut1, pGL-300-mut 2 and pGL-300-mut3 (Fig. 5J). The three mutant plasmids were transfected into 293T cells, and luciferase assays were performed. Figure 5K shows that the pGL-300-mut1 vectors decreased promoter activity compared to the wild-type control, indicating that the ETV5-binding site cluster is responsible for lncRNA CTBP1-DT transcription. A chromatin immunoprecipitation (ChIP) assay showed that approximately 16-fold enrichment of the promoter amplicons of lncRNA CTBP1-DT in the 0–300 bp binding sites in HGSOC cells was observed using the anti-ETV5 antibody (Fig. 5L), suggesting that ETV5 could directly bind to the promoter of lncRNA CTBP1-DT.

**Fig. 6:**
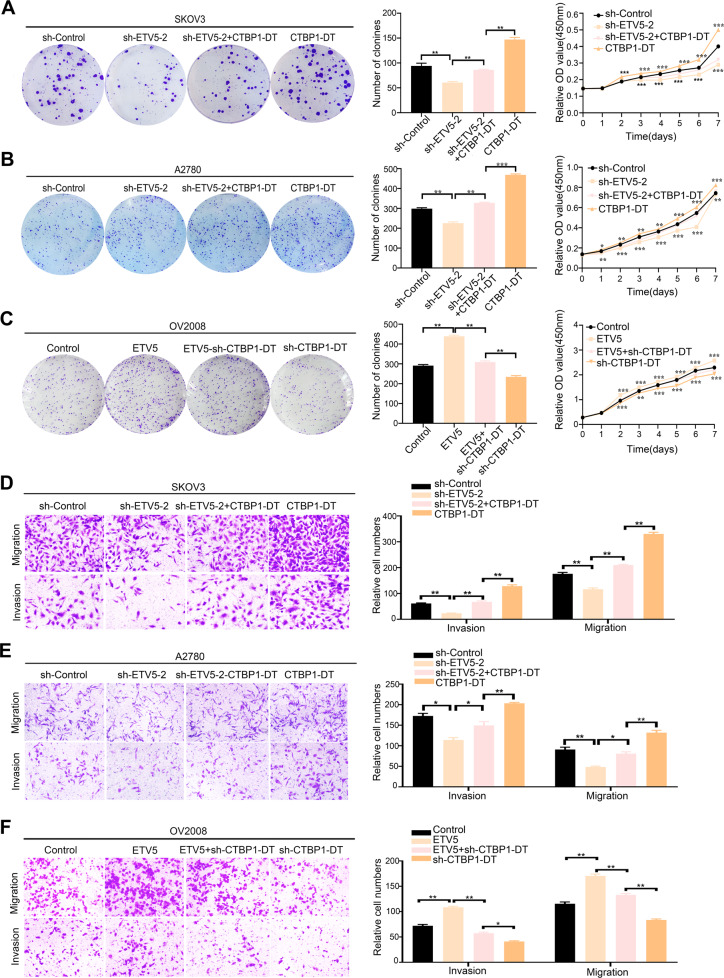
ETV5 enhances HGSOC cell proliferation, migration and invasion through lncRNA CTBP1-DT. **A**, **B** Cells were transfected with indicated sh-Control, sh-ETV5-2, sh-ETV5-2 with CTBP1-DT and CTBP1-DT, respectively. CCK-8 and colony formation assays were used to determine the proliferation of SKOV3 or A2780. **C** Cells were transfected with indicated Control, ETV5, ETV5 with sh-CTBP1-DT and sh-CTBP1-DT, respectively. Proliferation of OV2008 was detected by CCK-8 and colony formation assays. **D**, **E** After transfected with sh-Control, sh-ETV5-2, sh-ETV5-2 with CTBP1-DT and CTBP1-DT, transwell migration and invasion assays were performed in SKOV3 and A2780, respectively. **F** After transfected with Control, ETV5, ETV5 with sh-CTBP1-DT and sh-CTBP1-DT, transwell migration and invasion assays were performed in OV2008, respectively. Data are presented as the mean ± SEM. *P < 0.05, **P < 0.01, ***P < 0.001.

Next, we determined whether the effect of ETV5 overexpression or knockdown on lncRNA CTBP1-DT affected the malignant biological behaviour of HGSOC cells. Knockdown of ETV5 significantly decreased cell proliferation over a 7-day culture in SKOV3 and A2780 cells, which was reversed after overexpression of lncRNA CTBP1-DT. Consistent with the proliferation outcomes, silencing ETV5 by shRNA also decreased the colony-forming ability of both cell lines (Fig. 6A, B). In addition, the transwell assay revealed a clear decline in migratory capacity of SKOV3 and A2780 cells transfected with sh-ETV5-2. Moreover, the invasion assay using SKOV3 and A2780 cells revealed that ETV5 markedly promoted cell invasion compared with the empty vector pcDNA3.1 transfection. This effect was abolished by lncRNA CTBP1-DT co-transfection (Fig. 6D, E). Proliferation, migration and invasion assays revealed that the overexpression of ETV5 enhanced the malignant biological behaviour of OV2008 cells, which was reversed after knockdown of lncRNA CTBP1-DT (Fig. 6C, F). These findings demonstrated that ETV5-induced HGSOC cell proliferation, migration and invasion might have been due to the transcription of lncRNA CTBP1-DT.

To investigate whether ETV5 could be associated with MAP3K3 expression level in HGSOC, the relationship between them in HGSOC tissue was visualised (Supplementary Fig. 1). The expression of ETV5 was highly positively correlated with MAP3K3, which made our study more comprehensive.

## Discussion

Our data demonstrated upregulation of lncRNA CTBP1-DT in HGSOC tissues compared to normal fallopian tube tissues and relatively higher expression in ovarian carcinoma cell lines than in other types of tumour cell lines, such as oesophageal carcinoma, angiosarcoma and clear cell renal cell carcinoma cell lines. Among 76 HGSOC patients, those with overexpressed lncRNA CTBP1-DT had significantly shorter OS and DFS rates. Similarly, higher expression of lncRNA CTBP1-DT was associated with significantly reduced OS in the TCGA HGSOC datasets, which is consistent with our experiments.

The biological function of lncRNA CTBP1-DT is rarely known. After detecting the relative expression of lncRNA CTBP1-DT by qRT-PCR, we found that lncRNA CTBP1-DT had high expression in SKOV3 and A2780 and low expression in OV2008. Consequently, SKOV3 and A2780 were selected to perform a loss-of-function assay, while OV2008 was chosen for gain-of-function assays. In this study, the proliferative, migratory and invasive capabilities of SKOV3 were significantly suppressed by the downregulation of lncRNA CTBP1-DT. Correspondingly, these features were remarkably enhanced by the upregulation of lncRNA CTBP1-DT in OV2008. Furthermore, tumour weight and volume were significantly reduced following the knockdown of lncRNA CTBP1-DT in orthotopic xenograft mouse models. In the present study, we did not observe metastasis of the subcutaneously transplanted tumour. However, Zhang et al. intraperitoneally injected HGSOC cells into nude mice to form peritoneal metastases, and metastatic lesions were found in the mesentery, omentum and liver [23]. We speculate that the different injection methods could explain the absence of metastatic lesions of the transplanted tumour in the present study. These findings support the potential pro-oncogenic function of lncRNA CTBP1-DT in HGSOC. Abundant evidence showed that abnormal expression of lncRNAs could lead to the development and progression of various tumours, including ovarian carcinoma. LncRNA HEIH has been found to accelerate cell proliferation and inhibit cell senescence in OC [24]. Moreover, lncRNA SNHG17, HOTTIP and DSCR8 are independent prognostic factors associated with recurrence in OC [25–27], suggesting that lncRNAs have considerable prognostic potential in OC.

Expectedly, MAP3K3 was regulated by lncRNA CTBP1-DT in HGSOC progression. In SKOV3, the abundance of MAP3K3 mRNA and protein decreased or increased after knockdown or overexpression of lncRNA CTBP1-DT, respectively. Knockdown of lncRNA CTBP1-DT in SKOV3 inhibited cell proliferation, migration and invasion, which could be reversed by overexpression of MAP3K3. In addition, knockdown of MAP3K3 abrogated the effects of lncRNA CTBP1-DT overexpression in OV2008. These results indicate that MAP3K3 mediates the effect of lncRNA CTBP1-DT in regulating the malignant biological behaviour of HGSOC cells in vivo. Previous studies have confirmed that lncRNA USP2-AS1 [28], HOXD-AS1 [29] and HEIH [24] are upregulated in OC, which can, in effect, promote the progression and development of OC cells by upregulating the expression of downstream target genes. LncRNA CTBP1-DT has been shown to elevate ZNF217 expression in cervical cancer [14] and regulate phosphatase and tensin homologue to suppress OC cell proliferation [30]. Our study also showed that lncRNA CTBP1-DT could promote malignant biological behaviours of HGSOC cells by upregulating the expression of MAP3K3.

Previously, lncRNAs were thought to be biologically useless molecules arising from simple transcriptional ‘noise’ [31]. Emerging evidence reveals that lncRNAs participate in cellular biological and pathological processes. The function of lncRNAs is dependent on their subcellular localisation [32]. To further explore the regulatory molecular network of lncRNA CTBP1-DT with MAP3K3, cell cytoplasmic/nuclear fractionation and RNA fluorescence in situ hybridisation (FISH) assays suggested that lncRNA CTBP1-DT was preferentially localised in the cytoplasm. LncRNAs located in the cytoplasm could participate in gene regulation, including acting as a sponge of miRNAs to competitively bind to miRNAs and further relieve the suppression of miRNAs on their target genes [33]. Subsequently, through miRDB, Targetscan, miRTarBase and StarBase analysis, 14 miRNAs that can bind MAP3K3 were predicted. Based on data from the RNA22 and DIANA websites, miR-188-5p could bind both lncRNA CTBP1-DT and MAP3K3 in similar seed regions. This was confirmed by luciferase reporter and RIP assays, which demonstrated that miR-188-5p could directly bind to lncRNA CTBP1-DT and MAP3K3 3’UTR, respectively.

Recent studies have reported that miR-188-5p is a tumour suppressor and is significantly downregulated in various cancers, such as gastric [33], breast [34] and colorectal cancer [35]. In this study, miR-188-5p inhibitor promoted lncRNA CTBP1-DT expression in OV2008; however, miR-188-5p mimics inhibited lncRNA CTBP1-DT expression. Consequently, the expression of MAP3K3 was consistent with the change in lncRNA CTBP1-DT. Furthermore, miR-188-5p inhibitor upregulated the expression of lncRNA CTBP1-DT and MAP3K3, which could be significantly reversed by its mimics. These results suggest that lncRNA CTBP1-DT may act as a miR-188-5p sponge to protect MAP3K3 from degradation, thus increasing MAP3K3 expression in HGSOC.

However, the regulators involved in the abnormal expression of lncRNA CTBP1-DT in HGSOC cells remain unclear. Similar to protein-coding transcripts, the transcription of lncRNAs is subject to typical epigenetic-mediated and transcription factor-mediated regulation [36, 37]. Here, ETV5 showed the highest correlation with lncRNA CTBP1-DT based on data from JASPAR, an online bioinformatics software programme. The ETS family of transcription factors contributes to several physiological and pathological processes such as embryogenesis, wound healing and tumour progression [38]. In the present study, we demonstrated that overexpression of lncRNA CTBP1-DT in HGSOC cells could be activated when the transcription factor ETV5 binds to its promoter region. In addition, a clear decline in the proliferative, migratory and invasive capacity of A2780 and SKOV3 was observed when ETV5 was knocked down. This phenomenon was reversed by knockdown of ETV5 and overexpression of lncRNA CTBP1-DT. Moreover, ETV5 markedly promoted malignant cell behaviour in OV2008. This effect was abolished by the knockdown of the lncRNA CTBP1-DT. The effects of ETV5 on HGSOC cell proliferation, migration and invasion may partly be due to the lncRNA CTBP1-DT, which coincides with the functions of lncRNA CTBP1-DT. Finally, there was a high positive correlation between ETV5 and MAP3K3, which further proves that ETV5 regulates the expression of MAP3K3 by regulating lncRNA CTBP1-DT expression.

In our study, we have shown that lncRNA CTBP1-DT is regulated by the transcriptional factor ETV5, which is significantly upregulated in HGSOC, and can be used as a prognostic biomarker for HGSOC patients. It functions as a ceRNA that competitively binds miR-188-5p, upregulates MAP3K3 and promotes HGSOC cell proliferation, metastasis and invasion. These findings provide a new mechanism for understanding the development and progression of HGSOC, and lncRNA CTBP1-DT may be a potential candidate for the prevention and treatment of HGSOC.

## Materials and methods

### HGSOC patients and tissue specimens

A total of 87 HGSOC tissues and 35 normal fallopian tube tissues were obtained from HGSOC patients who underwent surgery at First Affiliated Hospital, School of Medicine, Shihezi University (Xinjiang, China) from 2005 to 2019. None of the patients received radiotherapy or chemotherapy before surgery. All tissue samples were immediately frozen in liquid nitrogen after resection from HGSOC patients and stored at −80 °C. This study used human carcinoma tissues and was approved by the Committee for Ethical Review of Research involving Human Subjects of First Affiliated Hospital, School of Medicine, Shihezi University. Informed consent was obtained from all patients.

### Cell lines and culture conditions

Human ovarian carcinoma cell lines (SKOV3, A2780, C13*, OV2008, HeyA8 and OVCA433), oesophageal carcinoma cells (109), angiosarcoma cells (ISO) and clear cell renal carcinoma cells (786–0) were cultured in RPMI-1640 medium (GIBCO, Carlsbad, CA, USA) containing 10% foetal bovine serum (FBS; Biological Industries), 100 U/mL penicillin and 100 mg/mL streptomycin (Solarbio, Beijing, China) in humidified air at 37 °C with 5% CO_2_. HEK-293T cells were cultured in Dulbecco’s Modified Eagle Medium (GIBCO) supplemented with 10% FBS, 100 U/mL penicillin and 100 mg/mL streptomycin in humidified air at 37 °C with 5% CO_2_. SKOV3, A2780, HeyA8, 109, 786–0, ISO and 293T cells were purchased from the Chinese Academy of Sciences Type Culture Collection (Shanghai, China). OV2008 and C13* cells were obtained from Prof. Benjamin K. Tsang (Ottawa Health Research Institute, Ottawa, Canada). OVCA433 cells were obtained from Dr Gang Chen (Department of Gynecology and Obstetrics, Tongji Hospital of Huazhong University of Science and Technology).

### Plasmid construction and transfection

For lncRNA CTBP1-DT or ETV5 overexpression, the full-length lncRNA CTBP1-DT or ETV5 cDNA was amplified and subcloned into pcDNA3.1. An empty vector was used as a negative control. The putative lncRNA CTBP1-DT promoter regions (−2000/0, −1200/0 and −300/0) were PCR-amplified from the genomic DNA and then inserted into the HindIII-NheI sites upstream of the firefly luciferase in the pGL3-Basic vector (Promega, Madison, WI, USA). All constructs were named based on the location of the promoter fragments relative to the transcription start site (TSS). MAP3K3 and sh-MAP3K3 plasmids were provided by Dr Yang (Baylor College of Medicine, USA). All plasmids were isolated using an E.N.Z.A Eno-free Plasmid DNA Mini Kit II (OMEGA, D6950-01, USA). An hsa-miR-188-5p mimic was used instead of miR-188-5p, a chemically modified antisense oligonucleotide (antagomir AMO-101) was used to inhibit miR-188-5p expression and a scrambled oligonucleotide (GenePharma) was used as a control. Cells were seeded in 6-well plates and cultured to 60–70% confluence before transfection. Lipofectamine^®^ 2000 reagent (Life Technologies, USA) was used as the transfection medium according to the manufacturer’s protocol.

### Bioinformatics methods

Potential lncRNA-binding sites of MAP3K3 were predicted by the TCGA database (https://genome-cancer.ucsc.edu/proj/site/hgHeatmap/). The Kaplan–Meier Plotter tool (http://kmplot.com/analysis/) was used to evaluate the association between MAP3K3 and the prognosis of OC patients. The lncRNA CTBP1-DT sequence was downloaded from the UCSC Genome Browser (http://genome.ucsc.edu/), from which the 2000-bp TSS upstream sequence was extracted. To identify putative transcription factors, the promoter sequence of lncRNA CTBP1-DT was submitted to the JASPAR programme (http://jaspar.genereg.net/).

### RNA extraction and qRT-PCR analyses

Total RNA was extracted from the tissues and cultured cells using the miRNeasy FFPE Kit and miRNeasy Mini Kit (QIAGEN, Germany) in accordance with the manufacturer’s instructions. Approximately 1 μg of total RNA was reverse transcribed to cDNA (complementary DNA) using a miScript II RT kit (QIAGEN, Germany), and qPCR was performed using a miScript SYBR Green PCR kit (QIAGEN, Germany). Comparative quantification was performed using the 2^−ΔCt^ method, with GAPDH as the endogenous control. Primer sequences used in PCR are listed in Supplementary Table 1.

### Cell proliferation and colony formation assays

For the cell proliferation assay, 24 h after transfection, tumour cells were seeded into 96-well plates and cultured for 6 days. In each well, 10 μL of Cell Counting Kit-8 solution (Dojindo, Tokyo, Japan) was added daily. Cell viability was evaluated by measuring the absorbance at a wavelength of 450 nm. For the colony formation assay, tumour cells (500 cells per well) were seeded into 6-well plates 24 h after transfection. After 10–12 days of incubation, the cells were fixed in methanol and stained with 0.1% crystal violet. The colonies were counted using Quantity One software (Bio-Rad, Hercules, CA, USA). All experiments were performed three times, and the mean was calculated.

### Cell migration and wound healing assays

Cells were seeded in the upper chamber of transwell plates (Corning, NY, USA) in a serum-free medium. FBS (10%) was added to the lower chamber of the transwell. Next, cells were transfected as described above for 48 h. For the invasion experiments, the upper chamber was covered with RPIM-1640 and Matrigel (BD Biosciences, San Jose, CA) mixture. Finally, cells on top of the chamber were removed with cotton swabs, while cells that went through the membrane (lower chamber) were stained with 0.1% crystal violet (Solarbio, Beijing, China), observed and counted under a microscope at 100× magnification.

### Isolation of cytoplasmic and nuclear RNA

Cytoplasmic and nuclear RNAs of cells were extracted and purified using the Cytoplasmic and Nuclear RNA Purification Kit (NORGEN) according to the manufacturer’s instructions.

### Fluorescence in situ hybridisation (FISH)

Cells were fixed in 4% paraformaldehyde for 15 min at room temperature and then permeabilised with 0.5% Triton X-100 for 15 min at 4 °C. Cells were incubated with digoxigenin (DIG)-labelled LncRNA CTBP1-DT/18S/U6 probe or Control-FISH probe mix for 4 h at 55 °C and briefly washed with 2× saline-sodium citrate six times for 5 min. Horseradish peroxidase-conjugated anti-DIG secondary antibodies (Jackson, West Grove, PA, USA) were used to detect the signal, and nuclei were counterstained with DAPI. Images were obtained using an Olympus confocal laser scanning microscope.

### Luciferase reporter assays

Human HEK 293T cells (2.0 × 10^4^) grown in a 96-well plate were co-transfected with 150 ng of either empty vector or miR-188-5p, ETV5, 50 ng of firefly luciferase reporter comprising 3’UTR of MAP3K3, wild-type or mutant MAP3K3/lncRNA CTBP1-DT fragment and 2 ng of pRL-TK (Promega, Madison, WI, USA) using Lipofectamine^®^ 2000 reagent (Life Technologies, USA to act as a negative control. Cells were harvested 48 h after transfection for luciferase assay using a luciferase assay kit (Promega, USA) according to the manufacturer’s protocol. Transfection was performed in triplicate.

### Chromatin immunoprecipitation (ChIP)

ChIP assays were performed using a ChIP kit (CST, USA) following the manufacturer’s instructions. Briefly, cells were crosslinked with formaldehyde and sonicated to an average length of 0–2000 bp. Immunoprecipitation was conducted with an anti-ETV5 antibody (orb69269, Biorby, UK) or IgG control. The precipitated DNA was amplified by RT-PCR. Primer sequences used in ChIP are listed in Supplementary Table 1.

### RNA-binding protein immunoprecipitation (RIP) assay

RIP assay A Magna RIP RNA-Binding Protein Immunoprecipitation Kit (Millipore, USA) was used to determine the relationship between lncRNA CTBP1-DT and miR-188-5p or ETV5. Antibodies used for the RIP assay included anti-AGO2 and control IgG (Millipore, USA), and the coprecipitated RNAs were used for cDNA synthesis and evaluated by qRT-PCR. Primer sequences used in PCR are listed in Supplementary Table 1.

### Western blot assay and antibodies

Cell protein lysates were separated by 10% SDS–polyacrylamide gel electrophoresis, transferred to 0.45 μm polyvinylidene difluoride membranes (Solarbio, Beijing, China) and incubated with specific antibodies. Autoradiograms were quantified by densitometry (Quantity One software; Bio-Rad). β-actin antibody (1:1000, IE9A3, Zhongshan Biotechnology, Beijing, China) was used as a control. Anti-MAP3K3 (1:1000) was purchased from Abcam (ab40756, Cambridge, UK).

### Tumour formation assay and tumour imaging in a nude mouse model

A total of 16 BALB/c nu/nu female mice of 4–6-week-old were divided randomly into two groups (8 mice/group). SKOV3 cells (5 × 10^6^ cells) with or without sh-lncRNA CTBP1-DT were suspended in 200 μL PBS and subcutaneously injected into each flank of 4–6-week-old BALB/c nu/nu female mice. The mice were sacrificed after 36 days, and the maximum (L) and minimum (W) length and weight of the tumours were measured. The tumour volume was calculated as ½LW^2^. The animal experiments were approved by the School of Medicine, Shihezi University Animal Care and Use Committee.

### Tumour imaging in vivo

SKOV3 with or without sh-lncRNA CTBP1-DT fluorescent lentivirus were injected into the subcutaneous tissue of 16 BALB/c nu/nu female mice. After 36 days, the nude mice were anaesthetised for in vivo optical imaging by IVIS spectrum pre-clinical in vivo imaging system.

### Statistical analysis

All the data of this experiment are the results of three independent repeated experiments. SPSS 22.0 software and GraphPad Prism 8.0 were used to analyse the data. Data are presented as the mean ± standard error of the mean for the experimental data conforming to the normal distribution, and *t*-test or one-way ANOVA was used to analyse the significant differences between different groups. Median and quartile ranges are reported for non-normally distributed experimental data, and Wilcoxon rank sum test was used to analyse the significant differences between different groups. OS was evaluated by Kaplan–Meier survival curves and compared using the log-rank test. The classification data, such as age, FIGO stage, chemotherapy response and ascites, were described by frequency and percentage. The relationship between lncRNA CTBP1-DT and the clinicopathological features were compared by the *χ*^2^ test. Univariate and multivariate Cox proportional hazard regression models were used to determine the effects of variables on survival. Inspection level: *α* = 0.05. *P < 0.05, **P < 0.01, ***P < 0.001. *P* < 0.05 was considered statistically significant.

## Supplementary information

Supplementary Table 1

Supplementary Figure 1

Supplementary Figure 1 legends

## Data Availability

The datasets generated and/or analysed during the current study are available from the corresponding author on reasonable request.
