# Supplementary material for: ETV5-mediated upregulation of lncRNA CTBP1-DT as a ceRNA facilitates HGSOC progression by regulating miR-188-5p/MAP3K3 axis
Source: Cell Death Dis. 2021 Dec 9;12(12):1146. doi: 10.1038/s41419-021-04256-9 (PMC8660778; doi:10.1038/s41419-021-04256-9)
Supplement: Supplementary file 1 — Supplementary Table 1 [file 41419_2021_4256_MOESM1_ESM.docx]

Supplementary Table 1: the information of various primers and

the information of ChIP and RIP primers

| Name | Primer direction | Sequence (5’- 3’) |
| --- | --- | --- |
| ETV5 | Forward | CAGCACACGGGTTCCAGTCAC |
|  | Reverse | TGGCAGTTAGGCACTTCTGAATCG |
| lncRNA CTBP1-DT | Forward | AAAGCTAGCCCGCCCACGTCAGCGCCTGG |
|  | Reverse | AAAGCGGCCGCTGGCAGTTTTCA GCGATTG |
| miR-188-5p | Forward | CATCCCTTGCATGGTGGAGG |
| MAP3K3 | Forward | CGAAAGTACACGCGGCAGAT |
|  | Reverse | CAGCAGAGTCTCGGAGGATGTT |
| GAPDH | Forward | GAGTCAACGGATTTGGTCGT |
|  | Reverse | TTGATTTTGGAGGGATCTCG |
| U6 | Forward | CTCGCTTCGGCAGCACA |
|  | Reverse | AACGCTTCACGAATTTGCGT |
| miR-188-5p- mimics |  | UAACAGUCUCCAGUCACGGCC |
| miR-188-5p- mimics-NC |  | UUCUCCGAACGUGUCACGUTT |
| miR-188-5p- inhibitor |  | GGCCGUGACUGGAGACUGUUA |
| miR-188-5p- inhibitor-NC |  | CAGUACUUUUGUGUAGUACAA |
