# Supplementary figures and images for: ETV5-mediated upregulation of lncRNA CTBP1-DT as a ceRNA facilitates HGSOC progression by regulating miR-188-5p/MAP3K3 axis
Source: Cell Death Dis. 2021 Dec 9;12(12):1146. doi: 10.1038/s41419-021-04256-9 (PMC8660778; doi:10.1038/s41419-021-04256-9)

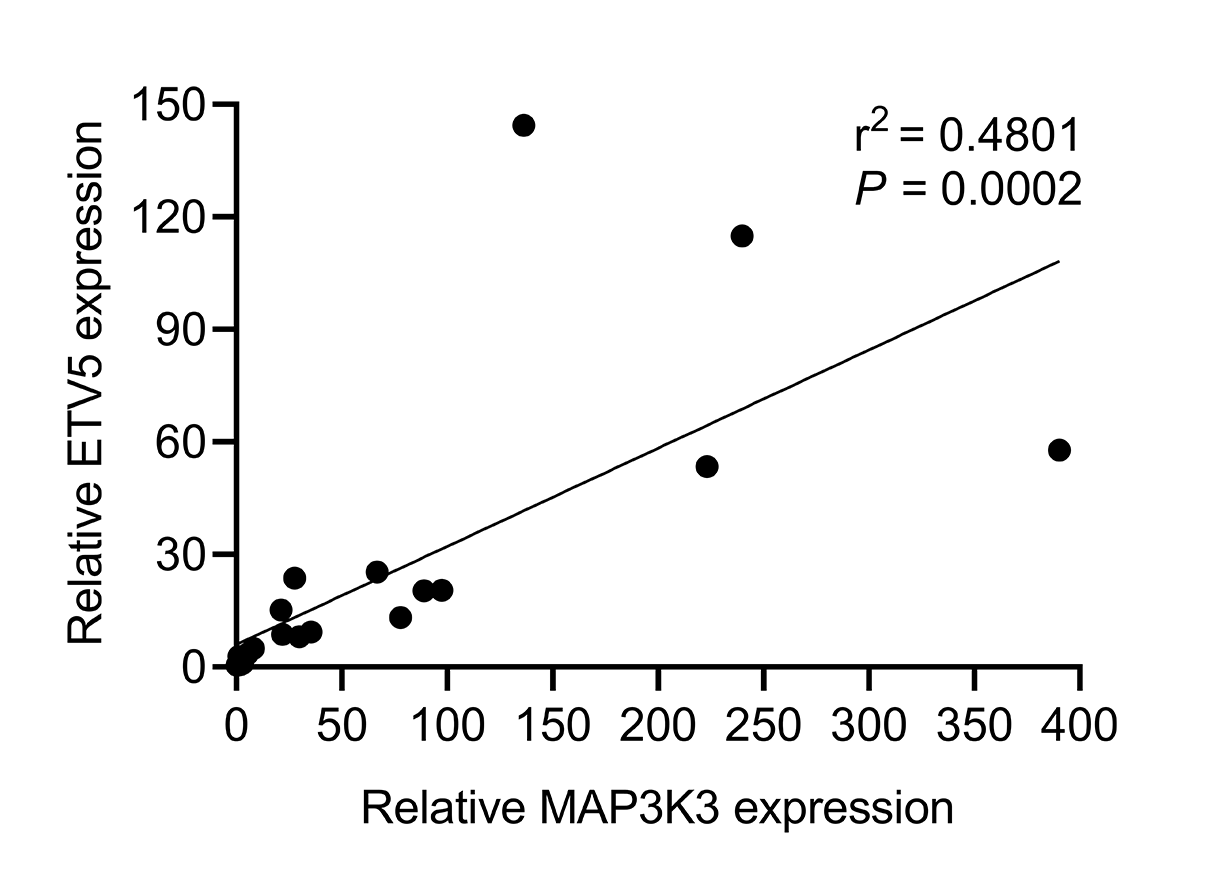

Supplement: Supplementary file 2 — Supplementary Figure 1 [file 41419_2021_4256_MOESM2_ESM.tif]
